# Supplementary material for: Predictors of early newborn deaths at Dar es Salaam public regional referral hospitals: A prospective observational hospital-based study
Source: PLoS One. 2025 Dec 11;20(12):e0338497. doi: 10.1371/journal.pone.0338497 (PMC12698010; doi:10.1371/journal.pone.0338497)
Supplement: Attachment — Submitted filename: Reviewers Comment PONE-D-25-05428.docx [file pone.0338497.s002.docx]

Reviewers Comment:

Thank you for the opportunity to review the manuscript “Predictors of Early Neonatal Mortality at Public Regional Referral Hospital in Dar es Salam Tanzania: A prospective observational hospital-based study.

General Overview: The proposal addresses important issues concerning child health in the public health space, specifically neonatal mortality and its associated predictors.

The Outline of the manuscript is in tandem with the journal.

**Abstract:**

Methods. The target population should be rephrased as all neonates in the hospital. The outcome is the death of neonates within 72 hours of life.

The phrase “Unless otherwise specified, an α-level of 5% was used as a limit of type 1 error rate in findings*” should be deleted from the material and methods in the abstract as it is a hanging tense”.*

Conclusion: The word *relatively* should be deleted from the sentence.

**Introduction:**

Well written, with the need to avoid some informal words in between tenses.

Rewrite/delete the tense, *for instance,* as: The three commonest factors associated with early neonatal mortality are…

Paragraph 2, line 3: Delete the word *“Antagonistic”. Please kindly explain the sentence, “However*, in some reported statistics, these factors are either contradictory or antagonistic, dependent on a number of mechanisms”

Methods:

What is the difference between the study population and the targeted population? I guess the target population is the author's choice when defining the study’s primary outcome.

The authors also need to clarify the participants enrolled. Were the participants only those delivered in the three referral hospitals, or did they include those referred from other hospitals for continued care in the three centres? This is important because there seems to be some conflicting tense of eligibility in the sample procedure sub-section and the recruitment and study duration sub-section.

The last sentence in the data collection procedure section appears redundant advise to delete it.

Variable Design and Measurement: If children delivered out of the referral facilities were involved in the study. Then, the place of delivery should be included as a variable.

Ethical clearance: Please do rephrase the tense: “that their babies will be subjected to minimal physical discomforts, especially during physical examinations as well as where to send their queries (if any) regarding the study/investigations in general” *to the babies will be subjected to routine physical examination on enrollment*

*What is the formula for computing the Early Neonatal mortality rate*

Results:

The results section is poorly written. It is just only a display of tables without phrases or tenses to describe the resulting output. The authors need to redo the result section significantly.

Example: Tabe 1:

The median age of participants enrolled in the study was 6 days, with a male-to-female ratio of (1:0.8). 71% of the participants were delivered via SVD, 26% via cesarean section

In table 1: Were the assisted delivery through SVD or C/S

Can the Place of delivery be better categorised? I presume all the hospital deliveries were delivered in the three primary referral facilities in the study, while the health centre, dispensary, home and others were out of the facilities. What does the other imply since it is only one subject?

Table 2: This table is confusing. The authors should help depict more clearly. The need for clear definitions of terms or diagnoses is important.

Where all the RDS associated with Prematurity or the MAS associated with Birth Aspyxia

Discussion:

Poorly written discussion.

The first paragraph appears more as postulations rather than the true summary of the findings from the results.

The assertion of residual factors seems unclear. What does the author mean by the term residual factors?
