# Supplementary material for: Predictors of early newborn deaths at Dar es Salaam public regional referral hospitals: A prospective observational hospital-based study
Source: PLoS One. 2025 Dec 11;20(12):e0338497. doi: 10.1371/journal.pone.0338497 (PMC12698010; doi:10.1371/journal.pone.0338497)
Supplement: Attachment — Submitted filename: Predictors of Early Neon Mort in Dar es Salaam Rev.docx [file pone.0338497.s003.docx]

TITLE: **Predictors of early neonatal mortality at public regional referral hospitals in Dar es Salaam, Tanzania: A prospective observational hospital-based study**

**REVIEW REPORT**

1. **Summary of the Research**

This paper presents a prospective observational hospital-based study to assess the rate and predictors of early neonatal mortality (within 72 hours of birth) in three regional referral hospitals in Dar es Salaam, Tanzania. Data were collected using a CRF and multivariable binary logistic regression was used to obtain predictors of mortality. Ethical approval was obtained from the Institutional Research Committee of the Hubert Kairuki Memorial University in Dar es Salaam, Tanzania, and mothers signed informed consent forms before recruitment of their children in the study.

Results showed that 370 neonates (within first 7 days of life) were recruited into the study between May and November 2023. The leading causes of death were birth asphyxia, preterm delivery, and respiratory distress syndrome, while the predictors of early neonatal death were respiratory rate, and gestational age.

The **strengths** of the paper are:

1. The authors’ interest in predictors of early neonatal mortality as identifying these predictors can enable the deployment of measures to ensure these vulnerable babies survive.
2. The study was based in regional reference hospitals serving the whole of the Dar-es-Salaam area and beyond.

The manuscript however, has some major issues which are highlighted.

**Introduction:** There many claims in the introduction which need further clarification/correction. Below are a few examples.

1. The WHO definition of early neonatal death is death occurring in the first 7 days of life, not the first 3 days.
2. The “demographic data on neonatal death risk factors” are said to be “controversial”, but neither the factors nor the controversy are stated/explained.
3. It is also stated that the 3 main factors associated with early neonatal deaths, viz, neonatal, maternal and hospital system factors can be either contradictory or antagonistic without any explanation/examples of the contradictions/antagonism

**Methods**

1. Babies were said to have been followed up for seven days or till death, whichever came first. Since the outcome of interest was mortality in the first 3 days, what was the significance of follow up beyond the first 3 days of life?
2. Recruitment: neonates born (and admitted) at study sites within 7 days of life as well as referrals from lower hospitals aged ≤ 7 days were included in the study. What data did those children admitted after 3 days of life contribute to the study outcome?
3. Data Management: Mortality pattern and immediate cause of death are said to be shown in a table and pie chart respectively, but these were not included in the results. The ICD-11 classification of underlying causes of death was not obvious.
4. Very importantly, there was no **definition of terms** used in the paper. The definition/explanation of terms would have greatly facilitated understanding of the work.

**Results**

1. The number of babies recruited was not explicitly stated, and follow up days are not a part of recruitment.
2. We infer that 370 children were recruited from the gender row of table 1. However, the number of children that died, the age at these deaths were not stated.
3. The Chi square analysis presented in table 2 is difficult to appreciate
4. The variables that are said to be significantly associated with mortality within the first 3 days of life (Respiratory rate (low, within normal range, or high?), gestational age (term, preterm, post term?), and study site are not well explained.
5. As a result of the foregoing, it is difficult to appreciate the stated “early neonatal” mortality rate of 278.1/1000 live births.

**Discussion**

1. The first sentence saying 1/3 of delivered babies in study settings died during follow up period is ambiguous: the number of babies born during the study was not stated, did the deaths referred to here occur within the first 3 days or the first 7 days, which was the follow up period?

**Comments:**

1. The study data should be thoroughly reviewed and the paper rewritten
2. Editorial assistance from someone more fluent in the English language may be useful

**Reviewer**

Dr Agatha N. David

Director of Research and Consultant Paediatrician

Clinical Sciences Department

Nigerian Institute of Medical Research (NIMR), Lagos, Nigeria
