# Supplementary material for: Predictors of early newborn deaths at Dar es Salaam public regional referral hospitals: A prospective observational hospital-based study
Source: PLoS One. 2025 Dec 11;20(12):e0338497. doi: 10.1371/journal.pone.0338497 (PMC12698010; doi:10.1371/journal.pone.0338497)
Supplement: Attachment — Submitted filename: PONE MS Reviewer recommendation.docx [file pone.0338497.s004.docx]

**Manuscript title:**

**Predictors of early neonatal mortality at public regional referral hospitals in Dar es Salaam, Tanzania: A prospective observational hospital-based study.**

**The authors are invited to address the section on “Data collection procedure” as follow:**

1. Data quality issues need attention. Clinical and laboratory data were collected on neonates from three hospitals hence three different research teams thus raising data quality assurance concerns. It is commendable that the instrument used to collect data was pilot-tested. However, there was no indication that the investigators were trained to use the tool. What did the authors do to address data consistency was assured? The study collected laboratory as well as clinical data. Did the Paediatric MDs (residents) attend all the deliveries and conducted the Apgar scoring or the midwives? Did all hospitals use the same (type, model) weighing scales and pulse oximeters for the babies? Did the authors use one lab or each hospital conducted their laboratory analysis (eg blood glucose)? Quality control measures for the clinical measurements and labs should be reflected in the write up.
2. Respiratory rate was one of the clinical parameters that predicted mortality as reported in this communication. In what way did the respiratory rate contribute to mortality? One would have expected that the pathological conditions that have bearing on the respiratory rate should have been factored into the analysis. Could this be reviewed and addressed?
