# Supplementary material for: Predictors of early newborn deaths at Dar es Salaam public regional referral hospitals: A prospective observational hospital-based study
Source: PLoS One. 2025 Dec 11;20(12):e0338497. doi: 10.1371/journal.pone.0338497 (PMC12698010; doi:10.1371/journal.pone.0338497)
Supplement: Attachment — Submitted filename: RESPONSE TO REVIEWERS.doc [file pone.0338497.s007.doc]

**RESPONSE TO REVIEWERS’ – PONE - D-25-05428**.

| **REVIEWERS’ QUERIES/RECOMMENDATIONS** | **SECTION**  **[PAGE NUMBER]** | **AUTHORS’ RESPONSE** | **CHANGES MADE**  **[YES/NO]** |
| --- | --- | --- | --- |
| 1. Abstract - Methods. The target population should be rephrased as all neonates in the hospital | Abstract  [pp. 2 ] | Indeed, in this clinical research, ‘target population’ was *neonates who died within the first 72-hours of delivery*.  All neonates in the hospitals were the study population and not the *target population*. | No |
| 2. The phrase “Unless otherwise specified, an α-level of 5% was used as a limit of type 1 error rate in findings*” should be deleted from the material and methods in the abstract as it is a hanging tense”.* | Abstract  [pp. 2] | Deleted completely as suggested by the reviewers. | Yes |
| 3. Conclusion: The word *relatively* should be deleted from the sentence | Abstract  [pp. 2] | Deleted completely as recommended by the reviewers | Yes |

| **REVIEWERS’ QUERIES/RECOMMENDATIONS** | **SECTION**  **[PAGE NUMBER]** | **AUTHORS’ RESPONSE** | **CHANGES MADE**  **[YES/NO]** |
| --- | --- | --- | --- |
| 4. The WHO definition of early neonatal death is death occurring in the first 7 days of life, not the first 3 days | Introduction  [pp.3] | In this research paper, we adopted the American Academy of Pediatrics definition of ‘early neonatal death’ that refers to death occurring in the first 72-hours (3-days) of life.  We have also included ‘definition of terms’ in the manuscript | Yes |
| 5. The “demographic data on neonatal death risk factors” are said to be “controversial”, but neither the factors nor the controversy are stated/explained | Introduction  [pp. 3] | We provided/stated the controversies associated with the term ‘early neonatal deaths’ in the introduction section.  *Specifically, we highlighted the differences in the definition of ‘early neonatal death’ as to what appears to be informally referring to ‘American vs European’ standards. We specified that there appears to be confusion in literature as whereas American Academy of Pediatrics considers ‘early neonatal period’ to be within the first 72-hours after delivery, in Europe (including WHO) definition, the same observation refers to the first seven days of life* | Yes |

| **REVIEWERS’ QUERIES/RECOMMENDATIONS** | **SECTION**  **[PAGE NUMBER]** | **AUTHORS’ RESPONSE** | **CHANGES MADE**  **[YES/NO]** |
| --- | --- | --- | --- |
| 6. It is also stated that the 3 main factors associated with early neonatal deaths, viz, neonatal, maternal and hospital system factors can be either contradictory or antagonistic without any explanation/examples of the contradictions/antagonism | Introduction  [pp. 3] | We have provided summary of the justifications behind both contradiction and antagonism.  Contradiction: the discrepancy in the definition of early neonatal period (death) between what appears to be ‘American vs European’ definitions.  Antagonism: differences in the causes of ‘early neonatal deaths’ between advanced economies vs. ‘low and middle income countries’ | Yes |
| 7. Babies were said to have been followed up for seven days or till death, whichever came first. Since the outcome of interest was mortality in the first 3 days, what was the significance of follow up beyond the first 3 days of life? | Methods  [pp. 5] | Investigators designed a research protocol on analyses for both deaths at day 3 and day 7 of life by design since even though the primary research question relied on ‘*incident neonatal mortality and associated factors at day 3*’ but there was a secondary research question that focused on analyzing ‘*incident neonatal mortality on day 7*’.  The original research paper will be on ‘early neonatal mortality and associated factors within 3 days of life’ and later on there will be another publication (secondary analysis!) for ‘incident neonatal mortality within 7 days of life’ | No |

| **REVIEWERS’ QUERIES/RECOMMENDATIONS** | **SECTION**  **[PAGE NUMBER]** | **AUTHORS’ RESPONSE** | **CHANGES MADE**  **[YES/NO]** |
| --- | --- | --- | --- |
| 8. What is the difference between the study population and the targeted population? I guess the target population is the author's choice when defining the study’s primary outcome | Methods  [pp. 5] | Target population is defined in a standard format as population to which the primary research findings/outcomes will be applicable. | No |
| 9. The authors also need to clarify the participants enrolled. Were the participants only those delivered in the three referral hospitals, or did they include those referred from other hospitals for continued care in the three centres? This is important because there seems to be some conflicting tense of eligibility in the sample procedure sub-section and the recruitment and study duration sub-section. | Methods  [pp. 5] | We only included newborns delivered at the study sites during this study period only. It was not clear where was the source of confusion to readers in the initial draft  Specifically, it is vividly written as   **Sampling procedure:** All newborns born at Amana, Mwananyamala and Temeke between 17 May and 16 November 2023 were eligible to be included into the study.  **Recruitment and study duration:** Newborns were recruited immediately after birth at the study sites. Recruitment was from 17^th^ May 2023 to (and including) 16^th^ November 2023. | No |

| **REVIEWERS’ QUERIES/RECOMMENDATIONS** | **SECTION**  **[PAGE NUMBER]** | **AUTHORS’ RESPONSE** | **CHANGES MADE**  **[YES/NO]** |
| --- | --- | --- | --- |
| 10. The last sentence in the data collection procedure section appears redundant advise to delete it. | Methods  [pp. 6] | Deleted as recommended by reviewers | Yes |
| 11. Variable Design and Measurement: If children delivered out of the referral facilities were involved in the study. Then, the place of delivery should be included as a variable. | Methods  [pp. 6] | We only included children born at the study sites in this study. | No |
| 12. What data did those children admitted after 3 days of life contribute to the study outcome? | Methods  [pp. 5] | They will contribute in subsequent analysis (secondary data analysis) that will be published in the near future. | Yes |
| 13. Data quality issues need attention. Clinical and laboratory data were collected on neonates from three hospitals hence three different research teams thus raising data quality assurance concerns.   It is commendable that the instrument used to collect data was pilot-tested. However, there was no indication that the investigators were trained to use the tool. | Methods  [pp. 6] | Data quality assurance concerns were kept at near zero possibilities since  1.The tool was pilot tested  2.Research team members underwent a formal 1-day ‘good clinical and ethics research practice’ workshops done during the time of tools’ pilot testing.  3.Since all the three regional referral hospitals are financed by the same donor sources, they normally have the same lab and imaging tools. We also conducted a quick test-retest reliability as well as content validity analyses of all test results with no resultant statistically significant differences in findings during pilot test. | No |

| **REVIEWERS’ QUERIES/RECOMMENDATIONS** | **SECTION**  **[PAGE NUMBER]** | **AUTHORS’ RESPONSE** | **CHANGES MADE**  **[YES/NO]** |
| --- | --- | --- | --- |
| 14. Very importantly, there was no **definition of terms** used in the paper. The definition/explanation of terms would have greatly facilitated understanding of the work | Methods  [pp. 5] | We have included a sub-section on ‘*definition of terms*’ in the methods section. For ease legibility, we decided to include it as the second sub-section, immediately after the study design section. | Yes |
| 15. Ethical clearance: Please do rephrase the tense: “that their babies will be subjected to minimal physical discomforts, especially during physical examinations as well as where to send their queries (if any) regarding the study/investigations in general” *to the babies will be subjected to routine physical examination on enrollment*   *What is the formula for computing the Early Neonatal mortality rate* | Methods  [pp. 7] | We have made the changes as per the recommendations by reviewers  We have also included the definition and formula for computing early neonatal mortality rate used in this analysis at the methods section of the manuscript. | Yes. |
| 16. The number of babies recruited was not explicitly stated, and follow up days are not a part of recruitment | Results  [pp. 8] | Number of babies recruited were explicitly stated in the manuscript using newborn-time of follow-up (see the first paragraph of the results section) since the study was prospective by design. In classical epidemiology, studies adopting prospective design report total recruitment using person-time of follow-up rather than an absolute number characteristic in cross-sectional studies. | No |
| 17. The results section is poorly written. It is just only a display of tables without phrases or tenses to describe the resulting output. The authors need to redo the result section significantly. | Results  [pp. 8-10] | Results section has been extensively re-written as depicted in the new submitted manuscript file | Yes |

| **REVIEWERS’ QUERIES/RECOMMENDATIONS** | **SECTION**  **[PAGE No.]** | **AUTHORS’ RESPONSE** | **CHANGES MADE**  **[YES/NO]** |
| --- | --- | --- | --- |
| 18. The first sentence saying 1/3 of delivered babies in study settings died during follow up period is ambiguous: the number of babies born during the study was not stated, | Discussion  [pp. 11] | We explicitly derived the statistic (nearly 1/3 of delivered babies dying) directly from the interpretation of the incident early newborn death statistic computed and summarized in this study findings.  Likewise, we also stated the number of babies born during the study using ‘person-time’ of follow-up since our study was prospective by design.  **Note;** in prospective studies, classic epidemiology entails summarizing the statistic of people followed-up using person-time’ and not an absolute count figure commonly stated in cross-sectional or retrospective studies! | No |
