# Supplementary material for: Predictors of early newborn deaths at Dar es Salaam public regional referral hospitals: A prospective observational hospital-based study
Source: PLoS One. 2025 Dec 11;20(12):e0338497. doi: 10.1371/journal.pone.0338497 (PMC12698010; doi:10.1371/journal.pone.0338497)
Supplement: Attachment — Submitted filename: Predictors of Early Neon Mort in Dar es Salaam Rev.docx [file pone.0338497.s006.docx]

TITLE: **Predictors of early neonatal mortality at public regional referral hospitals in Dar es Salaam, Tanzania: A prospective observational hospital-based study**

**REVIEW REPORT**

1. **Summary of the Research**

This paper presents a prospective observational hospital-based study to assess the rate and predictors of early neonatal mortality (within 72 hours of birth) in three regional referral hospitals in Dar es Salaam, Tanzania. Data were collected using a CRF and multivariable binary logistic regression was used to obtain predictors of mortality. Ethical approval was obtained from the Institutional Research Committee of the Hubert Kairuki Memorial University in Dar es Salaam, Tanzania, and mothers signed informed consent forms before recruitment of their children in the study.

Results showed that 370 neonates (within first 7 days of life) were recruited into the study between May and November 2023. The leading causes of death were birth asphyxia, preterm delivery, and respiratory distress syndrome, while the predictors of early neonatal death were respiratory rate, and gestational age.

The **strengths** of the paper are:

1. The authors’ interest in predictors of early neonatal mortality as identifying these predictors can enable the deployment of measures to ensure these vulnerable babies survive.
2. The study was based in regional reference hospitals serving the whole of the Dar-es-Salaam area and beyond.

**Comment**

The issues highlighted in the previous reviews of the manuscript have been satisfactorily addressed and the manuscript is recommended for publication.

**Reviewer**

Dr Agatha N. David

Director of Research and Consultant Paediatrician

Clinical Sciences Department

Nigerian Institute of Medical Research (NIMR), Lagos, Nigeria
