# Supplementary material for: Predictors of early newborn deaths at Dar es Salaam public regional referral hospitals: A prospective observational hospital-based study
Source: PLoS One. 2025 Dec 11;20(12):e0338497. doi: 10.1371/journal.pone.0338497 (PMC12698010; doi:10.1371/journal.pone.0338497)
Supplement: Attachment — Submitted filename: REPONSES TO REVIEWERS- PONE-D-25-05428R1.doc [file pone.0338497.s009.doc]

**REVIEWERS’ RESPONSES FOR MANUSCRIPT – PONE-D-25-05428R1**.

| **REVIEWERS’ ISSUES/QUERY/RECOMMENDATIONS** | **SECTION**  **[page number]** | **AUTHORS’ JUSTIFICATIONS/RESPONSE TO THE CHANGES/STATUS MADE** | **CHANGES MADE**  **[YES/NO]** |
| --- | --- | --- | --- |
| Neonatal death mortality were used in several sections of the write up. The authors either sticks to Neonatal deaths or mortality and not death mortality. | Title – [pp. 1]  Abstract – [pp. 2]  Introduction – [pp. 3-4]  Methods – [pp. 5-7]  Results – [pp. 8-10]  Discussion – [pp. 11-13] | -Authors wholeheartedly agreed to use the word ‘deaths’ instead of mortality for wider legibility of the intended message. | YES |
| ***Early neonatal death is defined by the American Academy of Paediatrics (AAP) as death in the first 72 hours of life***  This is incorrect; the AAP defines early neonatal death as death in the first 7 days of life, in line with the WHO (*Paediatrics 2016. 137(5): e20160551).* This was reaffirmed in December 2024*.* | Introduction - [pp. 3]  Methods – [pp. 5] | Authors accepted the standard definition as per the reviewers’ suggestion and hence had to change not only the definition of the term but also re-run the analyses that incorporated the former definition (early mortality ≤ 3 days – 72 hours) to a newer one of 7-days duration | YES |
| Some statements require references eg. "Besides, there is palpable evidence that Dar es Salaam city is among cities with highest neonatal deaths in Tanzania”. | Introduction – [pp. 3] | Missing reference that had to be added | YES |

| **REVIEWERS’ ISSUES/QUERY/RECOMMENDATIONS** | **SECTION**  **[page number]** | **AUTHORS’ JUSTIFICATIONS/RESPONSE TO THE CHANGES/STATUS MADE** | **CHANGES MADE**  **[YES/NO]** |
| --- | --- | --- | --- |
| ***The controversies are said to stem from the inconsistency between American and European/WHO definitions.***  This is debunked by the congruity of the two definitions as shown above. I believe they were misled by the disparities discussed in one of their referenced articles (Ref. 5), which disparities in neonatal mortality rate (NMR) across several European countries, comparing mortality rates and gestational-age associated mortality between the countries with the lowest NMR and the other countries. This should be understood in context and has nothing to do with the stated objective of this study.  In addition, the antagonism/contradiction among maternal, neonatal and health system factors in NMR is yet to be explained. These unexplored health system factors may actually be responsible for the so-called **residual factors** that they believe contributed to the high mortality rate. | Introduction – [pp. 3] | The entire sentences have been deleted after changing the definition of early neonatal period from 3 days (72-hours) to 7 days post-delivery. | YES |

| **REVIEWERS’ ISSUES/QUERY/RECOMMENDATIONS** | **SECTION**  **[page number]** | **AUTHORS’ JUSTIFICATIONS/RESPONSE TO THE CHANGES/STATUS MADE** | **CHANGES MADE**  **[YES/NO]** |
| --- | --- | --- | --- |
| The author would need to clarify some statements eg."Specifically, neonates born (and admitted) at study sites within 37 days of life were recruited into the study." I thought is about predictors of neonatal deaths occurring within 72 hrs of life | Methods – [pp. 5] | Authors changed the definition of early neonatal deaths from 3 days (72-hours) to 7 days. | YES |
| Follow up days beyond 3 days of life is said to be for another publication.  It should have been clearly stated that what was being reported in this manuscript is the 3-day NMR of a cohort of newborns followed up from birth to 7 days of life. | An answer to the previous reviews queries | Authors changed the definition of the concept ‘early neonatal period’/’early neonatal death’ from earlier 72-hours (3-days) to the current 7-days duration. The changes di not only affected the definition of terms but also changed even the analyses made by incorporating newborns up to 7 days. | YES |
| they said they only recruited babies born in the study hospitals.  This is refuted by this statement in the methods section: “Besides, we also recruited neonates who were referred/transferred from other lower hospitals to the study settings provided that they were ≤ 3 days old.” | Methods – [pp. 5] | Authors deleted that sentence and hereby declare that to have been an erroneous statement that was earlier refuted during protocol design. The error was introduced by one reviewer from IRB who directed investigators for those babies to be also included.  However, investigators rejected that idea during subsequent protocol submission for IRB since it was to affect the calculation of ‘*incident early neonatal mortality*’ to the extent that it would have rendered the calculation of that index impossible on statistical grounds. | YES |

| **REVIEWERS’ ISSUES/QUERY/RECOMMENDATIONS** | **SECTION**  **[page number]** | **AUTHORS’ JUSTIFICATIONS/RESPONSE TO THE CHANGES/STATUS MADE** | **CHANGES MADE**  **[YES/NO]** |
| --- | --- | --- | --- |
| ***“Number of babies recruited were explicitly stated in the manuscript”*** | Results – [pp. 8] | Authors added the total number of babies recruited | YES |
| The authors did not discuss the possible hospital system factors that may be responsible for neonatal deaths which may serve as predictors or are these part of the residual factors. | Discussion – [pp. 11] | Authors included ‘hospital/centre’ as one among potential HOSPITAL SYSTEM factors. Specifically, we separately analysed each hospital. Besides, we included each of the three hospitals separately in the multivariable binary logistic regression analysis whereby Temeke regional referral hospital was taken as a reference hospital under HOSPITAL SYSTEM factor (refer to table 3) | NO |
| The authors should highlight the possible residual factors responsible for neonatal deaths even if the study does not capture or what comprises the residual factors | Introduction – [pp. 3] | By default, the definition of *residual factors* included ‘latent variables…’ and by default could not be explicitly stated by investigators even though potentially statistically estimated.  We wish to request the reviewer to consider the term ‘residual factors’ as per the definition proposed in the introduction section of the manuscript. | NO |

| **REVIEWERS’ ISSUES/QUERY/RECOMMENDATIONS** | **SECTION**  **[page number]** | **AUTHORS’ JUSTIFICATIONS/RESPONSE TO THE CHANGES/STATUS MADE** | **CHANGES MADE**  **[YES/NO]** |
| --- | --- | --- | --- |
| Table 2. Factors should be changed to diagnoses. | Results [pp. 9] | Changed as per the reviewers’ recommendations | YES |
| "Otherwise, we do believe the hypothesized ‘residual factors for early neonatal mortality’ to have a potential for deleterious health consequences for newborns who managed to survive post-early"" What are these residual factors. The readers would like to know what makes up the residual factors and learn from it so as to provide better care for our babies | Discussion [pp. 12] | Authors kindly request the reviewer to refer to the proposed definition of the term ‘residual factors’ explicitly stated in the introduction section on page 3 of the manuscript document.  By default, we hypothesized that ‘residual factors’ to be *de facto latent variables*. *Latent variables* by their very nature cannot be stated though statistically can be estimated. | NO |
