# Supplementary material for: Predictors of early newborn deaths at Dar es Salaam public regional referral hospitals: A prospective observational hospital-based study
Source: PLoS One. 2025 Dec 11;20(12):e0338497. doi: 10.1371/journal.pone.0338497 (PMC12698010; doi:10.1371/journal.pone.0338497)
Supplement: Attachment — Submitted filename: Early Neon Mort in Tanz Rev 3.docx [file pone.0338497.s008.docx]

TITLE: **Predictors of early neonatal mortality at public regional referral hospitals in Dar es Salaam, Tanzania: A prospective observational hospital-based study**

**3^RD^ REVIEW REPORT**

The response of the authors to the issues raise in the 2^nd^ review have been noted and consisted of adopting the correct definition of early neonatal death (death in 1^st^ 7 days of life) and explicitly including the total number of babies followed up (370).

Other issues raised were not addressed:

- How many of the 370 admitted babies actually died: from table 2, we have a total of 175 deaths and 299 living (giving a total of 474). We are told that this is because some babies had multiple diagnoses (in the presence of multiple diagnosis, what was the primary cause of death?). However, if we took the total of 474, then 175 deaths would be 36.9%, which would be in agreement with what is reported in some literature of about a third of admitted neonates dying.
- How many live births occurred in the study hospitals during the 6 months of the study? This should serve as the denominator for the neonatal mortality rate in number/1000 live births.
- As stated in the previous reviews, it would have been easier to use the number of deaths over the 2,212 days of follow up to give the number of deaths /100 days of follow up of neonates admitted after birth.
- The tables are difficult to interpret; for table 2 for instance, what was the comparator for birth asphyxia that gave the values: ꭓ^2^ of 20.4, Df of 1, and p value of 0.000. Same difficulty for all the other diagnoses. Table 3 is even more difficult for me to interpret.
- A lot of the parameters said to be collected with the CRF, including maternal education, time of registration for ANC, number of ANC visits, maternal conditions like chorioamnionitis and antepartum haemorrhage (omitted were maternal hypertension and diabetes), cadre of health providers at delivery, whether babies were singletons or multiples, time to admission to neonatal unit, and so on were not included in the analysis. The addition of these parameters would have enriched the study.
- From the discussion, it appears the major interest of the authors are the *“residual factors of neonatal mortality”.* These unnamed and unknown factors are speculations at best and should properly be mentioned at the end of the discussion (not the beginning) as possible causes of the “high”mortality.
- I still maintain that it is not sufficient to mention “*residual factors which are not known but can be statistically measured”.* The autors should have explored deeper to discover the reason(s) for the discrepancies in deaths among the 3 study hospitals. The design of the study should have included possible health system factors such as: are the number of healthcare workers of different cadres adequate for the patient load, are there delays in patient admission, surgical procedures, are there patient fees that may hinder provision of care, are there adequate supply of materials at all times, are there unnecessary bottle-necks that hinder prompt patient care, etc.?

**Summary**

Many of the issues raised in the 2^nd^ review have not been satisfactorily addressed.

**Recommendation**

I believe those corrections are necessary not only to aid understanding of the study but also to contribute to knowledge.

**Reviewer**

Dr Agatha N. David

Director of Research and Consultant Paediatrician

Clinical Sciences Department

Nigerian Institute of Medical Research (NIMR), Lagos, Nigeria
