# Supplementary material for: Predictors of early newborn deaths at Dar es Salaam public regional referral hospitals: A prospective observational hospital-based study
Source: PLoS One. 2025 Dec 11;20(12):e0338497. doi: 10.1371/journal.pone.0338497 (PMC12698010; doi:10.1371/journal.pone.0338497)
Supplement: Attachment — Submitted filename: RESPONSES TO REVIEWERS - PONE-D-25-05428R2.doc [file pone.0338497.s011.doc]

**RESPONSES TO REVIEWERS’ FOR MANUSCRIPT – PONE-D-25-05428R2**.

| **Reviewers’ comments/queries/recommendations** | **Section**  **[Page number]** | **Authors’ justifications/responses to the changes/status made** | **Changes made**  **[Yes/No]** |
| --- | --- | --- | --- |
| 1. How many of the 370 admitted babies actually died: from table 2, we have a total of 175 deaths and 299 living (giving a total of 474). We are told that this is because some babies had multiple diagnoses (in the presence of multiple diagnosis, what was the primary cause of death?). However, if we took the total of 474, then 175 deaths would be 36.9%, which would be in agreement with what is reported in some literature of about a third of admitted neonates dying | Results  [pp. 9] | - Authors have re-written table no. 2 to incorporate the comparator values. Thus, the total number of newborns who died was 104 and vivid in the sum total of each reported diagnosis in the death column. We hope we have removed the unnecessary confusion to reviewer’s/readers via incorporating the comparator rows in table no. 2 - Indeed, it was erroneous to summate the **gross/total numbers of deaths** in table no. 2 due to existence of multiple diagnoses in some studied neonates.   e.g. about 45% of neonates who died of RDS on day 1 were also reported to have ***birth asphyxia*** immediately after birth | Yes |
| 2. How many live births occurred in the study hospitals during the 6 months of the study? This should serve as the denominator for the neonatal mortality rate in number/1000 live births | Results  [pp. 8] | - 370 live-newborns were delivered and followed-up at study settings during the study period.   **Note:**   1. We excluded by design all newborns admitted in the wards but delivered at lower facilities or home prior to admission.      2. We also excluded by design all stillbirth deliveries. | No |
| **Reviewer’s comments/queries/recommendations** | **Section**  **[Page number]** | **Authors’ justifications/responses to the changes/status made** | **Changes**  **made**  **[Yes/No]** |
| 3.As stated in the previous reviews, it would have been easier to use the number of deaths over the 2,212 days of follow up to give the number of deaths /100 days of follow up of neonates admitted after birth | Results  [pp. 8] | - Noted.   That is *near exactly* what authors did in estimating incident early neonatal death rates. However, instead of using number of deaths/100 days of follow-up of neonates admitted after birth, we rather estimated the early neonatal death rates by using *number of deaths that occurred among live-births delivered at the settings in 2212 days of follow-up* over (divided by) *total number of live-births delivered at the study settings in 2212 days of follow-up*. Thereafter, the resultant estimate was multiplied by a factor of 1000 to get the estimated early neonatal death rates per 1000-live births   - Authors did not understand the source of the 100-days of follow-up parameter in the computation of early neonatal deaths rates. | No |
| 4. The tables are difficult to interpret; for table 2 for instance, what was the comparator for birth asphyxia that gave the values: ꭓ^2^ of 20.4, Df of 1, and p value of 0.000. Same difficulty for all the other diagnoses. Table 3 is even more difficult for me to interpret. | Results  [pp. 9 ] | - Noted and changed as per the reviewer’s advise/recommendations.   Specifically, in each neonatal diagnosis, a comparator row has been added for ease observation and calculation of both *observed* and *expected* values in the chi-square statistics. Thus, even though there are no changes in the overall numerics/message, authors considered it logical to include comparator row.   - Likewise, authors have made substantial changes to table 3 in order to facilitate easy understanding to readers. Specifically,  1. For all categorical variables that incorporated dummies, a reference category has now been included in the table. 2. Besides, a description on why some variables in table 3 are without reference values (they were treated as continuous variables) has also been justified in text. | Yes |

| **Reviewer’s comments/queries/recommendations** | **Section**  **[Page number]** | **Authors’ justifications/responses to the changes/status made** | **Changes**  **made**  **[Yes/No]** |
| --- | --- | --- | --- |
| 5. A lot of the parameters said to be collected with the CRF, including maternal education, time of registration for ANC, number of ANC visits, maternal conditions like chorioamnionitis and antepartum haemorrhage (omitted were maternal hypertension and diabetes), cadre of health providers at delivery, whether babies were singletons or multiples, time to admission to neonatal unit, and so on were not included in the analysis. The addition of these parameters would have enriched the study | Results  [8 - 10] | - Authors have included parameters that were initially left out in the results section. They are currently justified as text either before or as numerics in table 1 and either before as text or as numerics in table 3. - Besides, the justifications behind why some factors described in table 1(baseline characteristics) still did not appear in the final analysis has been made in text for readers/reviewers.   i.e. We included only those (maternal, neonatal or hospital-based) factors that satisfied the assumptions statistically associated with linear model fitting. Thus, for a factor to be included into the final multivariable binary logistic regression model, it had to show evidence of its residuals to be *normally distributed*, *homoscedastic* with neither significant *multi-collinearity*, *auto-correlation* nor *deviation from linearity* assumptions. Besides, for inclusion into the final multivariable model, its p-value must have been < 0.2 at univariate/crude statistics. | Yes |

| **Reviewer’s comments/queries/recommendations** | **Section**  **[Page number]** | **Authors’ justifications/responses to the changes/status made** | **Changes**  **made**  **[Yes/No]** |
| --- | --- | --- | --- |
| 6. From the discussion, it appears the major interest of the authors are the *“residual factors of neonatal mortality”.* These unnamed and unknown factors are speculations at best and should properly be mentioned at the end of the discussion (not the beginning) as possible causes of the “high”mortality. | Discussion  [pp. 11] | -Agreed and removed everything about ‘residual factors of neonatal mortality’ to the end of the discussion section as per reviewer’s recommendations/advice | Yes |
| 7. I still maintain that it is not sufficient to mention “*residual factors which are not known but can be statistically measured”.* The autors should have explored deeper to discover the reason(s) for the discrepancies in deaths among the 3 study hospitals. The design of the study should have included possible health system factors such as: are the number of healthcare workers of different cadres adequate for the patient load, are there delays in patient admission, surgical procedures, are there patient fees that may hinder provision of care, are there adequate supply of materials at all times, are there unnecessary bottle-necks that hinder prompt patient care, etc.? | Discussion  [pp. 11- 13] | -Agreed and some changes made. For instance, we included the comparator/reference hospital (for comparison) in the final analysis. However, some suggested changes were not considered since:   1. All the studied hospitals are in the same hierarchy in the Tanzania’s referral health system, and being managed/supervised by the same central ministry of health, and therefore their supplies are replicate equally resources at the study settings. i.e. Amana, Mwananyamala and Temeke hospitals have all the same number of child health specialists, the same hospital protocol as well as following the same national guidelines – e.g. in Tanzania, all children under five years of age (including neonates) receive free service on a *de jure* basis. Thus, exploring anything that is already guaranteed by laws (and order) would attract penalties from the ethics review (IRB) boards. 2. Some of the unnecessary ‘bottlenecks’ have been hypothesized and acknowledged by authors as study limitations.   e.g. there are potential indications that ‘early neonatal deaths’ in Dar es Salaam hospitals to have ‘seasonal variation’. The fact that our study was designed for 6-months only and made to be observational by design, means exact mechanisms about causal factors were far beyond the objectives and design of this observational study. | Yes. |
