# Supplementary material for: Predictors of early newborn deaths at Dar es Salaam public regional referral hospitals: A prospective observational hospital-based study
Source: PLoS One. 2025 Dec 11;20(12):e0338497. doi: 10.1371/journal.pone.0338497 (PMC12698010; doi:10.1371/journal.pone.0338497)
Supplement: Attachment — Submitted filename: Early Neon Mort in Tanz Rev 4.docx [file pone.0338497.s010.docx]

TITLE: **Predictors of early neonatal mortality at public regional referral hospitals in Dar es Salaam, Tanzania: A prospective observational hospital-based study**

**3^RD^ REVIEW REPORT**

The response of the authors to the issues raise in the 3^rd^ review have been noted.

However, significant issues consistently raised in previous reviews are yet to be addressed.

- The result of early neonatal mortality rate (NMR) of **281/1000** live births cannot be correct. ***In the introduction, the authors stated that the crude early neonatal mortality rate in Tanzania was 18/1000 live births, and the under-five mortality for Tanzania as at 2020 was 48/1000 live births!*** Sub-Saharan Africa has the highest NMR globally at 27/1000 live births. An alarming NMR of 281/1000 live births from anywhere in the world would result in a global address by the WHO. Researchers must be circumspect to accurately reporting study findings.
  - The authors simply divided the number of deaths (104) by the number of admitted, i.e. sick neonates (370) and got 0.281 which they multiplied by 1000 to get 281/1000.
  - This is however not to be reported as deaths /1000 live births. I am quite certain that in the 6-month period of the study there were far more than 370 births in these 3 referral hospitals and most of those births would have been live births. If the authors divided the number of deaths (104) by the total number of live births in the study sites (which was not reported) then they could have talked of deaths/1000 live births.
  - From their results what they have is that 28.1% of admitted sick babies in the early neonatal period died. This figure is similar to what ahs been reported in other studies.
- As stated in the previous reviews, it would have been easier to use the number of deaths over the 2,212 days of follow up to give the number of deaths /100 days of follow up of neonates admitted after birth.
- While table 2 has been corrected, Table 3 remains a challenge. In talking about associations, it is crucial to explain what is associated with what in order to provide clinical clarity. For instance, respiratory rate is said to be significantly associated with mortality; It is not stated whether high respiratory rate (tachypnoea), low respiratory rate (bradypnoea) or normal respiratory rate is the significant association, and in what way is the association, is it positive or negative? Similarly, gestational age is significantly associated with mortality; the question is what gestational age? It is universally understood that shorter gestational age, resulting in prematurity is associated with a higher likelihood of death. Is that what the authors found? This is not clearly reported. They could have divided gestational age into different groups e.g., preterm (<37 weeks), term (37-41 weeks) and post term (≥ 42 weeks); or early preterm (may be <32 weeks) versus term (37-41 weeks).
  - There was some explanation in the discussion but this is not enough. The results should be clear.
- They explained away the non-use of maternal and health system information said to have been collected in the CRF by using fancy statistical terms but they should find a way to incorporate and analyze all that data. The addition of these parameters will enrich the study.
- I still maintain that it is not sufficient to mention “*residual factors which are not known but can be statistically measured”.* The authors should have explored deeper to discover the reason(s) for the discrepancies in deaths among the 3 study hospitals. The design of the study should have included possible health system factors such as: are the number of healthcare workers of different cadres adequate for the patient load, are there delays in patient admission, surgical procedures, are there patient fees that may hinder provision of care, are there adequate supply of materials at all times, are there unnecessary bottle-necks that hinder prompt patient care, etc.?

**Summary**

Many of the issues raised in the 3 review have not been satisfactorily addressed.

**Recommendation**

It does not appear that the authors are willing to understand the salient points raised in the reviews. I therefore recommend that the manuscript be rejected as it should not be published as it is.

**Reviewer**

Dr Agatha N. David

Director of Research and Consultant Paediatrician

Clinical Sciences Department

Nigerian Institute of Medical Research (NIMR), Lagos, Nigeria
