# Supplementary material for: Predictors of early newborn deaths at Dar es Salaam public regional referral hospitals: A prospective observational hospital-based study
Source: PLoS One. 2025 Dec 11;20(12):e0338497. doi: 10.1371/journal.pone.0338497 (PMC12698010; doi:10.1371/journal.pone.0338497)
Supplement: Attachment — Submitted filename: RESPONSES TO REVIEWERS - PONE-D-25-05428R4.doc [file pone.0338497.s013.doc]

**RESPONSES TO REVIEWERS’ FOR MANUSCRIPT – PONE-D-25-05428R4.**

| **Reviewers’ comments/queries/recommendations** | **Section**  **[Page number]** | **Authors’ justifications/responses to the changes/status made** | **Changes made**  **[Yes/No]** |
| --- | --- | --- | --- |
| 1. The result of early neonatal mortality rate (NMR) of **281/1000** live births cannot be correct. | Results  [pp. 8 ] | We have made changes as per the reviewers’ recommendations/suggestions. We deleted the incident mortality figures and computed the prevalence of mortality among sick admitted newborns. Specifically, the sentence now reads as   “Besides, we also found the prevalence of deaths among followed-up babies to be 28.1% at the study sites during the study period” | Yes |
| 2. While table 2 has been corrected, Table 3 remains a challenge. In talking about associations, it is crucial to explain what is associated with what in order to provide clinical clarity. | Results  [ pp. 10 ] | We have made changes as suggested/advised by the reviewer.  Specifically, we categorized respiratory rates into 3 distinct groups namely tachypnea (RR> 60 b/min), eupnoea (RR of 40 – 60 b/min) and bradypnoea (RR < 40 b/min) and reported their estimates separately in each category  Likewise, for gestational age at delivery, we also categorized into three different groups and report their estimates accordingly. | Yes |

| **Reviewers’ comments/queries/**  **recommendations** | **Section**  **[Page number]** | **Authors’ justifications/responses to the changes/status made** | **Changes made**  **[Yes/No]** |
| --- | --- | --- | --- |
| 3. They explained away the non-use of maternal and health system information said to have been collected in the CRF by using fancy statistical terms but they should find a way to incorporate and analyze all that data. The addition of these parameters will enrich the study | Results  [pp. 9 ] | We fitted a newer binary multivariable logistic regression model that was considered PARSIMONIOUS enough (with LR-goodness of fit χ2 test score of 4.61 with 4 degrees of freedom) that was considered to be linear fit for our predictor variables. However, all efforts to include those factors were rendered futile since:   1. Some failed on linearity test (e.g. birth weight) – **TOLERATED but proved to be FUTILE in its predictive power (see table 3 on pp. 10)**. 2. Some had significant multicollinearity with the fitted linear model (e.g. number of available neonatologists, presence/absence of emergency neonatal resuscitation equipments) – **NON-TOLERABLE as no LINEAR model could be estimated with precision at any level of significance tested**! 3. Others displayed significant autocorrelation (e.g. time of delivery, birth order of the baby) - **NON-TOLERABLE as no fitted linear model was possible**. 4. And yet others displayed significant *heteroscedasticity* (e.g. history of maternal fever, chorioamnionitis and antepartum haemorrhage) with the outcome variable (neonatal death = 1) – **NON-TOLERABLE as they would render the fitted model without sensible estimates!**   Thus, whereas TOLEARANCE could be granted for VIOLATION of linearity assumption (i.e. birthweight) the rest were considered A NON-NEGOTIABLE for inclusion into the linear model as for sure they were to render the entire analysis useless due to EXTREME VIOLATION of linear model assumptions. And even though we decided to tolerate violation of linearity in order to confer reviewer’s recommendations, any sensible reader would be able to see that in table 3 – results section, since the resultant p-value on the univariate analysis were a unit (p-value = 1.000) unlike a maximum of 0.2 that were justifiably a threshold for model inclusion into the multivariable analysis. Thus, predictive power of the variable “birthweight” was almost NIL for early neonatal mortality in the fitted linear model (see table 3 on pp. 10)! | Yes |
