# Supplementary material for: Predictors of early newborn deaths at Dar es Salaam public regional referral hospitals: A prospective observational hospital-based study
Source: PLoS One. 2025 Dec 11;20(12):e0338497. doi: 10.1371/journal.pone.0338497 (PMC12698010; doi:10.1371/journal.pone.0338497)
Supplement: Attachment — Submitted filename: Predictors of Early Neon Mort in Dar es Salaam Final Rev.docx [file pone.0338497.s012.docx]

TITLE: **Predictors of early neonatal mortality at public regional referral hospitals in Dar es Salaam, Tanzania: A prospective observational hospital-based study**

**2^ND^ REVIEW REPORT**

The response of the authors to the issues raised in the initial review has been noted:

1. ***Early neonatal death is defined by the American Academy of Paediatrics (AAP) as death in the first 72 hours of life***

This is incorrect; the AAP defines early neonatal death as death in the first 7 days of life, in line with the WHO (*Paediatrics 2016. 137(5): e20160551).* This was reaffirmed in December 2024*.*

2. ***The controversies are said to stem from the inconsistency between American and European/WHO definitions.***

This is debunked by the congruity of the two definitions as shown above. I believe they were misled by the disparities discussed in one of their referenced articles (Ref. 5), which disparities in neonatal mortality rate (NMR) across several European countries, comparing mortality rates and gestational-age associated mortality between the countries with the lowest NMR and the other countries. This should be understood in context and has nothing to do with the stated objective of this study.

In addition, the antagonism/contradiction among maternal, neonatal and health system factors in NMR is yet to be explained. These unexplored health system factors may actually be responsible for the so-called **residual factors** that they believe contributed to the high mortality rate.

3. *Follow up days beyond 3 days of life is said to be for another publication.*

It should have been clearly stated that what was being reported in this manuscript is the 3-day NMR of a cohort of newborns followed up from birth to 7 days of life.

4. ***Recruitment:***
i. ***they said they only recruited babies born in the study hospitals.***

This is refuted by this statement in the methods section: “Besides, we also recruited neonates who were referred/transferred from other lower hospitals to the study settings provided that they were ≤ 3 days old.”

ii. ***“Number of babies recruited were explicitly stated in the manuscript”***

The above statement by the authors is not true. The number of babies recruited was not stated, and one only infers that it is 370 from the statement that majority were male (200 [57%]). Person-time follow up periods can be used in analysis and expressing some results but does not replace the total number of participants followed up even in prospective cohort studies. Also rates in such time-person series are given in events/specific time, e.g., deaths per 100 person days/months/years. In addition, the study population included babies surviving beyond 3 days as they were the comparison group. Their details were not included in this manuscript as they should have been.

5. ***Mortality Rate***

The humongous mortality rate of **278.1/1000 live births** should have given the authors pause. Even the under-five mortality rate in Africa in 1990 was less than this. There is a difference between deaths/1000 live births and deaths among admitted sick neonates. In the case of the former the total number of live births in the facilities should be the denominator and the children that died the numerator. It appears that this was a study of newborns admitted to the neonatal unit for various reasons. The recorded high death rate is therefore not so surprising. The study in Mauritania cited by the authors clearly stated that the study was among admitted neonates, 37% of whom died.

6. ***Mortality pattern:*** this was not clear from this paper. It would have enriched the paper to know the proportion that died on the first, second, and third days, the clinical association with the deaths on these days as this would have given insight into areas that may need attention. From Table 2, it appears that factors which are globally associated with neonatal mortality such as prematurity and birth asphyxia, were more common in children who survived beyond 3 days, and only respiratory distress syndrome (associated with prematurity!) was found to be significantly associated with early neonatal death”.
7. Finally, health system factors should be explored in subsequent studies or in the write-up of the 7-day follow up paper. These include description of the study sites in terms of bed space, number of deliveries and neonatal admissions, number and cadre of staff in the neonatal units, access to these facilities, health insurance/out of pocket/free services, time to implementation of clinical decisions such as caesarean deliveries etc.

**Summary**

There appears to be a lack of understanding of the subject matter by the authors.

**Recommendation**

The data should be reviewed and the manuscript rewritten.

**Reviewer**

Dr Agatha N. David

Director of Research and Consultant Paediatrician

Clinical Sciences Department

Nigerian Institute of Medical Research (NIMR), Lagos, Nigeria
